# Why has the epidemiology of RSV changed during the COVID-19 pandemic?

**DOI:** 10.1016/j.eclinm.2023.102089

**Published:** 2023-07-06

**Authors:** Bahaa Abu-Raya, Marina Viñeta Paramo, Frederic Reicherz, Pascal Michel Lavoie

**Affiliations:** a Department of Pediatrics, University of British Columbia, Vancouver, Canada; b British Columbia Children's Hospital Research Institute, Vancouver, Canada

**Keywords:** RSV, Resurgence, Waning immunity, COVID-19, Co-infection

## Abstract

The coronavirus disease 2019 (COVID-19) pandemic has drastically perturbed the epidemiology of Respiratory Syncytial Virus (RSV) respiratory tract infections in children. The reasons for this are not clear. In this article, we review the current literature and critically discuss the different theories to explain why the epidemiology of RSV has changed during the COVID-19 pandemic. Proposed mechanisms include decreased viral immunity in vulnerable age groups caused by the prolonged lack of RSV circulation early in the pandemic, potential Severe Acute Respiratory Syndrome Corona Virus 2 (SARS-CoV-2)-induced immune dysregulation, viral interactions between SARS-CoV-2 and RSV, and modifications in health-seeking behaviors as well as heath systems factors. Research in viral genomics and phylogeny, and more robust immunology research is needed to guide RSV prevention and health care resource planning.

## Introduction

Respiratory Syncytial Virus (RSV) is a leading cause of acute respiratory tract infections in young children, with an estimated 30 million cases, 3·6 million hospital admissions and 100,000 deaths each year, worldwide.^1^ Infants younger than six months are at higher risk of severe disease, particularly those born prematurely, with chronic lung or congenital heart disease, and children with neurological conditions or an immunodeficiency. Yet, healthy term-born infants contribute the highest to morbidity burden, with about one to two percent who need hospitalization across jurisdictions.^2^ RSV typically causes bronchiolitis, characterized by respiratory distress, cough and varying degrees of hypoxia due to airway congestion in older children, and might also present as apnea in young infants.^3^ Newborns are relatively protected against severe disease by maternal antibodies transferred via the placenta.^4^ However, these antibodies wane rapidly, with a peak in vulnerability to RSV around one to two months of age.^5^ Most children have been infected by RSV by their first or second birthday.^6^ Adults, on the other hand, have strong cellular immunological memory and thus, are often asymptomatic after contracting RSV, although they are still an important reservoir for viral transmission.^7^ In adults with chronic conditions or those older than 65 years, RSV can cause severe disease manifesting as pneumonia or bronchitis.^8^

Before the Coronavirus Disease 2019 (COVID-19) pandemic, detection of RSV infections followed a predictable seasonal pattern each year.^9^ During the winter months, RSV epidemics have been consistently peaking in temperate northern regions of the globe.^10^ Similarly, in tropical and subtropical regions, RSV transmission peaks during rainy seasons.^11^ Since humans are the only known reservoir of RSV, viral transmission is largely dependent on seasonal changes linked to environmental, demographic and human behavioral factors such as travelling, indoor gathering, local population density, outdoor temperature, and humidity.^11^, ^12^, ^13^, ^14^, ^15^ Seasonal RSV epidemics follow a biennial oscillation pattern, possibly driven by ecological factors, and a population-level immunity against RSV that is likely relatively short-lived, and in the range of six and 12 months, based on epidemiological models.^10^^,^^16^

## Changes in RSV epidemics associated with the COVID-19 pandemic

In the first year of the COVID-19 pandemic, soon after February–March 2020 when non-pharmaceutical interventions (NPI) were aggressively implemented, RSV cases immediately plummeted worldwide.^17^, ^18^, ^19^, ^20^, ^21^ As these measures were gradually relaxed, many areas of the world experienced different degrees of off-season resurgence of cases.^18^^,^^22^ Australians were among the first to experience an atypical surge in RSV cases, during their summer, between December 2020 and February 2021. Interestingly, they also reported a shift towards older children presenting to a medical clinic with symptomatic infections.^19^^,^^23^ Remarkably, this resurgence of RSV cases was unequally experienced across the country, with peaks in Western Australia between October first-December 12th of 2020, and the Melbourne area reaching the peak of cases around February ninth of 2021.^24^^,^^25^ Subsequently, other areas of the world also reported resurgences of cases, with tremendous interregional variability in intensity and clinical severity.^21^^,^^26^, ^27^, ^28^, ^29^, ^30^, ^31^, ^32^, ^33^, ^34^ The purpose of this article is to review the current evidence and discuss the different theories supporting the reasons why the epidemiology of RSV has changed during the COVID-19 pandemic.

## Waning of RSV immunity at the population level, or the immunity debt theory

To explain the atypical inter-seasonal resurgence of respiratory infections around the world, the Pediatric Infectious Disease Group proposed the concept of an “immunity debt”.^35^ According to this theory, the lack of immune stimulation by viruses for prolonged periods may have increased the pool of immunologically vulnerable individuals, specifically children, resulting in increased disease severity upon the resurgence of viruses with the relaxing of NPI. Supporting this theory, infants and women of childbearing age showed a decline in neutralizing RSV antibody titers in the Vancouver metropolitan area, over a one-year period of extremely low RSV detection in Canada, between May–June 2020 and February–June 2021.^36^ In contrast, no decrease in RSV T cell immunity was detected in adults over the same period.^36^ Decreased RSV antibody levels were also reported in a Dutch population, during a period of low viral detection, between June 2020 and February–June 2021.^37^ These data suggest that antibody-mediated immunity against RSV may have decreased, resulting in decreased maternal antibody protection at birth, accumulation of immunologically naïve children younger than 2 years of age who are at greater risk due to a delayed primary exposure to the virus, and increased vulnerability in children also due to a waning of RSV immunity in absence of repeated viral exposures. While an increasing pool of vulnerable children seems plausible, we lack evidence supporting other mechanisms proposed by the Pediatric Infectious Disease Group,^35^ related to a potential lack of innate immune training or an imbalance of the intestinal microbiota (in relation to the hygiene theory). Thus, until these concepts can be substantiated with more data, it may be more exact to refer to decreased RSV immunity at the population level, rather than an “immunity debt”.

## Potential interaction between SARS-CoV-2 and RSV

Another theory is that the circulation of multiple respiratory viruses during the resurgence period may have resulted in a high level of interactions between viruses, including an increase in viral co-infections or super-infections.^38^ Regarding potential interactions between viruses, these can have both additive or subtractive effects, as supported by observations from experimental models, clinical cases and/or epidemiological data.^38^ Indeed, infection with one virus could enhance or suppress replication of another virus in hosts, through structural or functional changes in the respiratory mucosa, such as, for example, induction of interferon responses.^38^ In the case of SARS-CoV-2 and RSV, available evidence from experimental models do not support that a SARS-CoV-2 infection or co-infection enhances RSV cytopathogenicity in a human bronchial epithelium cell model.^39^^,^^40^ Moreover, SARS-CoV-2 triggered weaker anti-viral innate immune responses in cell cultures compared with RSV as measured by interferon-stimulated genes.^39^

Generally, epidemiological studies have reported a relatively low prevalence of SARS-CoV-2 and RSV co-infection which also does not support the theory that prior or concurrent SARS-CoV-2 has led to more detection of RSV or more severe disease.^41^ A study from the UK, that included 6965 patients admitted with SARS-CoV-2 between February sixth, 2020, and December eight, 2021, and on whom tests for respiratory viral co-infections were recorded, found co-infections with RSV or influenza in 3·1% and 3·2% of cases, respectively,^42^ similar to a three percent rate of co-infection between RSV and SARS-CoV-2 found in a US study.^43^ A retrospective study of respiratory pathogens identified by the Johns Hopkins Diagnostic Laboratory in the US, between December 2019 and October 2021, found no cases of SARS-CoV-2 and RSV co-infection, among 37 RSV cases during August–October 2021.^44^ In another US study, a single center retrospective review of laboratory-confirmed viral respiratory cases during a surge of RSV between March 2021 and August 2021 found that only 1·4% of RSV cases had co-infection with SARS-CoV-2 (4/276).^45^ A recent US study of children admitted with SARS-CoV-2 infection between March 2020 and February 2022 reported seven percent of co-infection with RSV in cases where non-SARS-CoV2-2 testing was performed.^41^ In Italy, a retrospective analysis of samples collected from patients with acute respiratory syndrome in 2019–2021 showed no cases of SARS-CoV-2 and RSV co-infection despite that RSV was detected in 12·8% of cases.^46^ In Canada, preliminary analyses of all RSV cases in patients younger than 18 years at our institution at the British Columbia Children's Hospital found only 1·8% (16/880) and 1·7% (11/637) documented SARS-CoV-2 co-infection in during the 2021–2022, and 2022–2023 RSV seasons, respectively.^47^ In view of these observations, co-infections may appear relatively too infrequent to explain the excess hospitalizations and intensive care admissions in children, though that could explain a small proportion of the excess of cases during the resurgence.

## Immune dysregulations following SARS-CoV-2 infection

Others have proposed that SARS-CoV-2 infections may have induced immune dysregulation in children, increasing their susceptibility to other respiratory viruses, such as RSV. A small study showed that 15 patients who recovered from COVID-19 had lower percentage of un-switched memory B cells, and lower CD19 expression upon B cell receptor stimulation of B cells compared with healthy controls 10–12 weeks after infection.^48^ In another study, 69 patients who recovered from COVID-19 showed lower proportions of CD4 T and CD8 T cells, but higher proportions of (CD19+) B cells and (CD56+) NK cells at 12- and 16-weeks post-infection, compared with healthy controls matched by sex and age.^49^ Another study reported increased expression of activation and exhaustion T and B cell markers in patients hospitalized for COVID-19, and similar changes in non-hospitalized convalescent patients up to 45 days after infection, compared to healthy controls.^50^ Yet, in another study, COVID-19 convalescent patients had fewer neutrophils, higher expression of cytotoxic CD4 and CD8 T cell (e.g., HLA-DR and CD38), and B cell markers ten weeks after disease onset compared with heathy controls.^51^ Furthermore, COVID-19 patients more than two months after recovery and compared with unexposed individuals had significant decreases in frequencies of invariant NKT and NKT-like cells, a significant decrease in cytotoxic potential of T cells and NKT-like cells and a significant increase in significant regulatory T cell frequencies.^52^ Patients with long COVID-19 also showed highly activated innate immune cells, elevated expression of type I and type III IFN and a lack of naive T and B cells eight months after infection.^53^ Altogether, these data suggest that this immune dysregulation may persist months after following a recovery from COVID-19 with both potentially protective and deleterious effects on the immune system. It is important to point out that these immune dysregulation theories merit more investigations, but are essentially based on laboratory observations that is lacking direct support from clinical or epidemiological data. Therefore, the clinical relevance of these observations is still unclear. Perhaps one of the most compelling arguments against a SARS-CoV-2-mediated immune suppression can be drawn from the experience of New Zealand. Indeed, the lifting of the strict border closure policy in April 2021 in this country was followed by a massive increase in RSV hospitalizations and ICU admissions in children younger than four years. However, at the time this happened while the country had been still largely free of COVID-19 (the authors report one hospitalization and no ICU admissions due to COVID-19 before this period).^54^ The Australian experience of a large off-season resurgence of RSV cases at the end of 2020—beginning of 2021, before SARS-CoV-2 had significantly spread among children also argues against an immune dysregulation.^55^^,^^56^

## Increased RSV virulence

It remains possible that conditions linked to the COVID-19 pandemic may have favored the emergence of more transmissible or more virulent RSV strains although current data do not support this. RSV sequences from symptomatic patients during the surge in November 2022 in the US showed that 90% of the cases were caused by RSV-A while the remaining 10% were caused by RSV-B.^57^ Importantly, phylogenetic comparison of 40 RSV-A genomes suggested that the surge was not driven by a single lineage, but consisted of at least 9 distinct clades, all with time-to-most recent common ancestors dated between 2014 and 2016.^57^ Moreover, phylogenetic analysis of three RSV-B genomes also yielded a time-to-most recent common ancestors in 2016. These data suggest that the emergence of a single, highly transmissible RSV lineage is unlikely to have been responsible for the resurgence in 2022, and argue against selection of more virulent RSV strains during the pandemic. In another study from the US, genome analyses suggested that RSV-A and RSV-B lineages circulated during the 2021–2022 and 2022–2023 outbreak have been seen globally for almost a decade.^58^ Recently, a study from China reported amino acid substitutions in RSV BA9, including A269V, T274A, and T300I, between October 2020 and October 2021, compared to pre-pandemic periods. However, this was not seen for RSV A and the clinical significance of these substitutions is unclear.^59^ In Austria, RSV A and RSV B subtypes circulating during 2021 and 2022 surge belonged to lineages that were present prior COVID-19.^60^

## Other potential factors

Beside host and viral factors, it is important to consider that other factors that may have contributed to the changing epidemiology of RSV cases, including interregional and temporal variations in testing practices, individuals' behaviors, as well as societal and health system factors. In the early to middle stages of the pandemic, the volume of pediatric emergency department visits drastically decreased.^61^, ^62^, ^63^, ^64^, ^65^, ^66^, ^67^, ^68^ Unfortunately, during that period, children's access to primary care was curtailed, resulting in decreased routine immunization against vaccine-preventable respiratory infections.^69^^,^^70^ Changes in health-seeking behaviors may have also resulted in delayed consultations due to specific guidance from health authorities who sought to curtail the transmission of SARS-CoV-2, or to perfect patient flow and avoid overcrowding in pediatric emergency departments early on.^71^^,^^72^ In addition, it is likely that parents of young children may have never experienced seasonal respiratory illnesses in their infants, which could have increased the propensity for seeking medical care once cases began to resurge. Similarly, deteriorated primary care resources may have also resulted in a heightened tendency to pivot towards tertiary care centers for consultation, fueled by a media coverage of pediatric hospital crises over the last year, as respiratory cases resurged. Indeed, during the pandemic, many health care systems have experienced gaps in primary health care, compounded by health provider burnouts, and more research is needed to document the impact of the pandemic on health systems in general. This, and the lack of experience from early career health workers who also may have similarly less experience in a respiratory illness in a child, may have contributed to stress health systems further. The impact of these behavior changes on the respiratory illness crisis over the last year and whether these behavioral changes may have persisted up until now requires urgent studies. It is also unclear whether these factors may have sustainably changed individuals' approach towards respiratory tract infection prevention at the society level, delaying “normalization” of the epidemiology of RSV as experienced prior to COVID-19. All these questions certainly merit more research to orient effective solutions and stabilized frazzled health systems.

## Conclusion and future directions

More research is warranted to understand why the epidemiology of respiratory infections has changed since the COVID-19 pandemic. The prolonged lack of viral exposure and decrease in social contacts have likely increased the pool of vulnerable children and adults. Robust research is needed to understand the interplay between the different factors, including viral genomics and phylogenic, host immunological and epidemiological factors. Understanding these factors is important to help predict and anticipate the need for health resources for the upcoming season(s). The possibility that SARS-CoV-2 infections may have caused an immune dysregulation in children is worth investigating, but is in contradiction with epidemiological observations in New Zealand and Australia. Although we lack evidence to support changes in RSV virulence, fully understanding this will be crucial for the next seasons.

Understanding the factors driving the epidemiology of respiratory viruses has clinical implications. In the upcoming years, it will be important to continue to monitor RSV outcomes, and their impact on pediatric health care resources over longer consecutive periods, especially with the imminent arrival of long-acting RSV monoclonal antibodies and pregnancy vaccination.^73^ It will also be important to monitor how population-based interventions may impact the epidemiology and genetic of the virus, and to make sure these interventions remain effective in a changing viral landscape. To do so, we need to take advantage of all existing sources of data, and expand the use of novel viral detection methods, such as wastewater-based surveillance methods.^74^ In addition to enhancing our understanding of the virus, this will help refine the accuracy of prediction models,^75^ until pre-pandemic levels of RSV immunity and transmission are restored.

## Outstanding questions

The following questions remain outstanding: i) what will happen with RSV cases in the upcoming seasons (e.g. will the situation stabilizes or will it continue, and for how long)?—This will require ongoing monitoring of cases of RSV, but also other respiratory infections, with robust clinical severity outcome measures; ii) why these changes happened? A close monitoring of a diverse array of RSV immune outcomes in adults and children of different ages would help support an immune theory; more robust immunological and epidemiological studies are required, incorporating clinical outcomes following common respiratory infections between people previously infected with SARS-CoV-2, versus uninfected ones. Perturbations in seasonal RSV epidemics also raise important questions about how this will impact the therapeutic benefit of recently-approved RSV vaccines, and long-acting monoclonal RSV antibodies in young children. Thus, it will be particularly important also to monitor outcomes following these interventions. Finally, we should not assume that the crisis engendered by the recent changes in RSV epidemics were only due to immunological or viral factors, and more studies are needed to understand how the pandemic has affected the resilience of health systems, and other systemic factors.

**Search strategy and selection criteria**

We identified references for this review through searches of PubMed for articles published in English from March first 2020 to March first 2023 using the four search criteria: First criteria: “Respiratory Syncytial Virus” AND “Resurgence” OR “Outbreak” AND “COVID-19” AND “Immunity”; Second criteria: “SARS-CoV-2” OR “COVID-19” AND “immunodeficiency” OR “immune dysregulation” OR “immune dysfunction”; Third criteria: “health behavior” AND “covid 19” and “caregivers”; Fourth criteria: “Respiratory Syncytial Virus” AND “Transmission” OR “Epidemics” OR “Resurgence” AND “COVID-19”. Articles resulting from these searches and the relevant references cited in those articles were reviewed. We also searched the websites of Lancet journals, Journal of American Medical Association, The Journal of Infectious Diseases, Clinical Infectious, The Morbidity and Mortality Weekly Report, New England Journal of Medicine for published articles on RSV from March first 2020 to April first 2023. Relevant reports from the American Academy of Pediatrics and the United States Centers for Disease Control and Prevention were also included. An updated literature search was conducted on June fourth, 2023.

## Contributors

BA, MV, FR and PML conceptualized the article theme, searched and critically reviewed the relevant literature, wrote the article and approved its final version. All authors read and approved the final version of the manuscript.

## Declaration of interests

BA received honoraria for participation in live meetings from Sanofi Pasteur France and Canada related to pertussis (for BA) and RSV products, but not related to this study. PML received honoraria for participating in an Advisory Board meeting for Sanofi Canada, unrelated to this study. Other authors have no conflicts of interest to declare.
